# ‘The Choice of Places to dwell in’: Lord Bacon's controlled experiments on ‘putrefaction’ and ‘the Disposition of the Aire’ (pre-1626)

**DOI:** 10.1177/09677720251382305

**Published:** 2025-10-07

**Authors:** Max Cooper, Sarah Cooper

**Affiliations:** 1 Department of Primary Care and Public Health, 12190 Brighton and Sussex Medical School, Brighton, UK; 2 Department of Neurology, 156800 Royal Sussex County Hospital, Brighton, UK

**Keywords:** Francis Bacon, forensic medicine, housing, credentialing, post-mortem interval, decomposition, public health, Shakespeare, pathology, general practice

## Abstract

**Introduction:**

Lord Francis Bacon's (1561–1626) was fascinated by the phenomenon of ‘putrefaction’, which he saw in ‘moulds’ on food, ‘mosse…of the Earth, and Trees’ and the process of disease in living creatures. By observing the development of mould, Bacon deduced that certain ‘aires’ and ‘seats’ (places) were more healthy than others.

**Aim:**

To interpret Bacon's ideas about putrefaction.

**Method:**

Qualitative examination of Bacon's texts, especially ‘Sylva Sylvarum’.

**Results:**

Bacon proposes comparing the speed of putrefaction between pieces of ‘raw flesh’ of the ‘same Kinde and Bignesse’ in different settings: ‘within Doores’ and ‘abroad [outside]’ as well as ‘some height above the Earth’ and ‘upon the Flat [i.e. surface] of the Earth’. Thus, Bacon sought to identify more healthy ‘seats of dwelling’ …‘for [residential] Lodges, and Retiring Places for Health’.

**Conclusion:**

Bacon's experiments represent early landmarks in two fields of medical research: evidence-based public health measures to improve housing and (by calculating ‘post-mortem interval’) forensic pathology. These contributions appear to have been overlooked, despite informing Sir John Pringle's 1752 treatise on military medicine. Bacon called for ‘new learning’ and his forensic approach highlights a need to engender the ‘medical detective’ in modern students of medicine.

## Introduction

Francis Bacon (1561–1626), First Viscount St Albans and First Lord Verulam, is renowned for his contribution to the development of the modern scientific method. Bacon (see Figure 1) was a lawyer, Member of Parliament and served as Lord High Chancellor of England. His biography^1^ and wider contributions to medicine^2–4^ are considered in detail elsewhere. This article examines Bacon's pioneering footsteps into the fields of public health and forensic medicine which took the form of experiments on ‘putrefaction’ and the quality of ‘aires’. Given Bacon's rallying call to reform learning, the implications of his experiments and his wider ‘forensic approach’ to gathering evidence is then discussed with regard to modern medical education.

**Figure 1.**
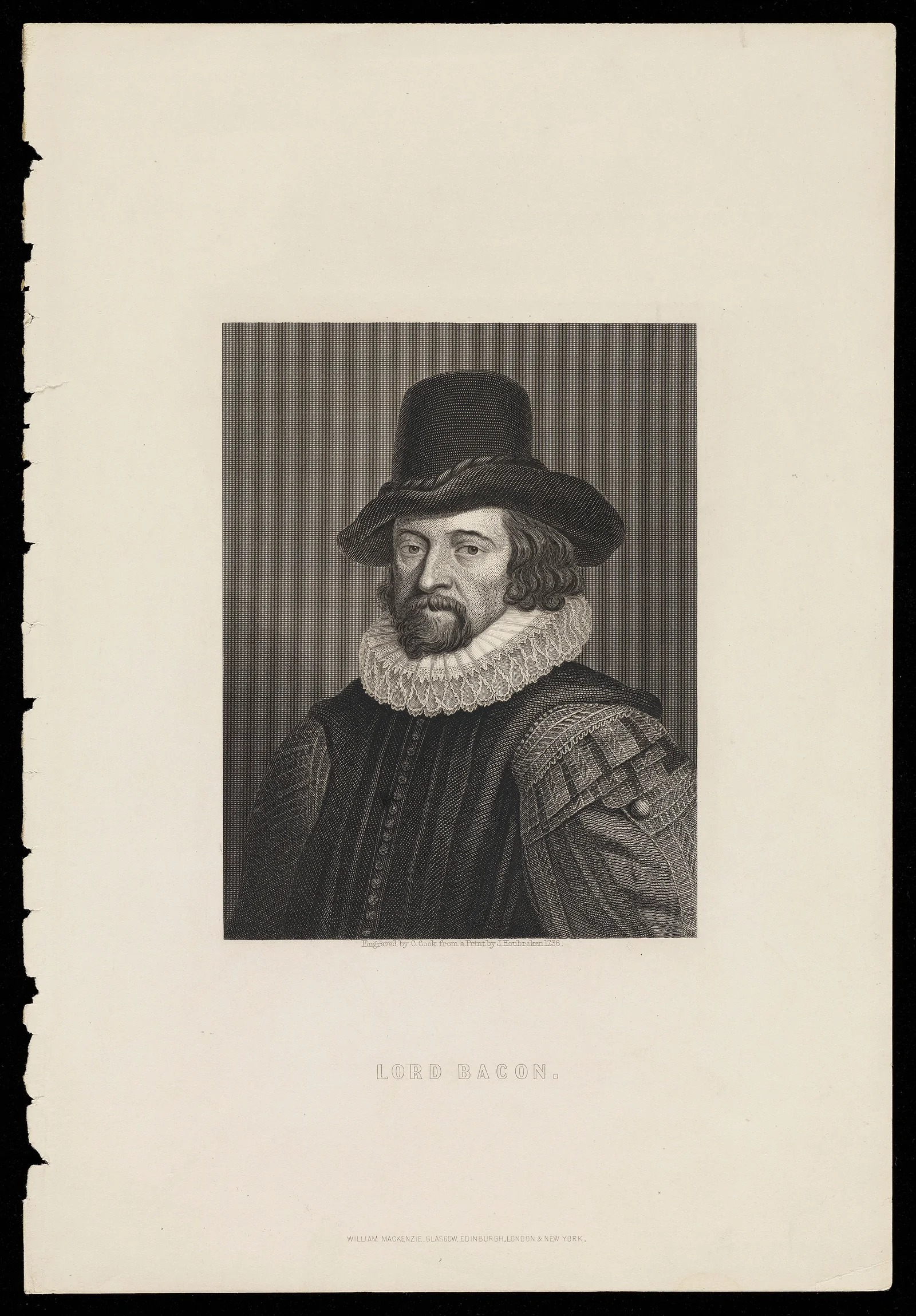
Francis Bacon, Viscount St Albans. Engraving by C. Cook after J. Houbraken, 1738. Image courtesy of the Wellcome Collection.

## The ‘Enducing and Accelerating of Putrefaction’

One of Bacon's unrecognised (or, rather, long-forgotten) contributions to medical research lies in brief controlled experiments described in his book ‘Sylva Sylvarum: Or, a Naturall Historie’.^5^ This book was first published in c1628 (i.e. after Bacon's death) by William Rawley ‘one of his Majesties Chaplaines’.^5^ This article draws upon its Fifth edition of 1639.^4^ Bacon considers the importance of air quality and season of the year as well as their effect on disease. Central to his enquiry is Bacon's understanding of ‘putrefaction’. This he sees as the opposite of ‘generation’ of life, i.e. a process of decay:

“The Enducing and Accelerating of Putrefaction, is a subject of a very Universall Enquiry: for Corruption is a Rociprocall to Generation: And they Two, are as Natures two Termes or Bundaries; And the Guides to Life and Death”.^5^ ^p. 73^

Bacon saw ‘putrefaction’ as a detrimental metamorphosis which took place in a broad range of substances: in ‘moulds’ on food, ‘mosse… of the Earth, and Trees’, in the transformation of wine into vinegar, the process of disease in living creatures,^5^ ^p. 75^ and even ‘Rust of Metalls’.^5^ ^p. 73^ He considered the addition of ‘watry moisture’^5^ ^p. 74^ as one key factor in the genesis of ‘putrefaction’. In such ways, ‘spirits’ could lead to ‘putrefaction of humours’ as happened in ‘Agues’,^5^ ^p. 74^ i.e. fevers.

The cause of putrefaction was perceived to be transmitted by ‘aire’, a conclusion seemingly based upon his observation of seeding in moulds and plants. In Bacon's time, most diseases were ascribed to bad ‘aire’. This had its roots in ancient Greek medicine, for example, Hippocrates’ book ‘On Airs, Waters and Places’ from circa 400 BCE^6^ (See Figure 2). Bacon notes:

“Putrefaction is the worke of the Spirits of Bodies, which ever are unquiet to Get forth, and Congregate with Aire… there followeth Digestion, or Maturation… Which dissolveth the Consistence of the Body into much Inequalitie; as in Flesh, Rotten Fruits, Shining Wood, &c. And also in the Rust of Metalls…”^5^ ^p. 73^

**Figure 2.**
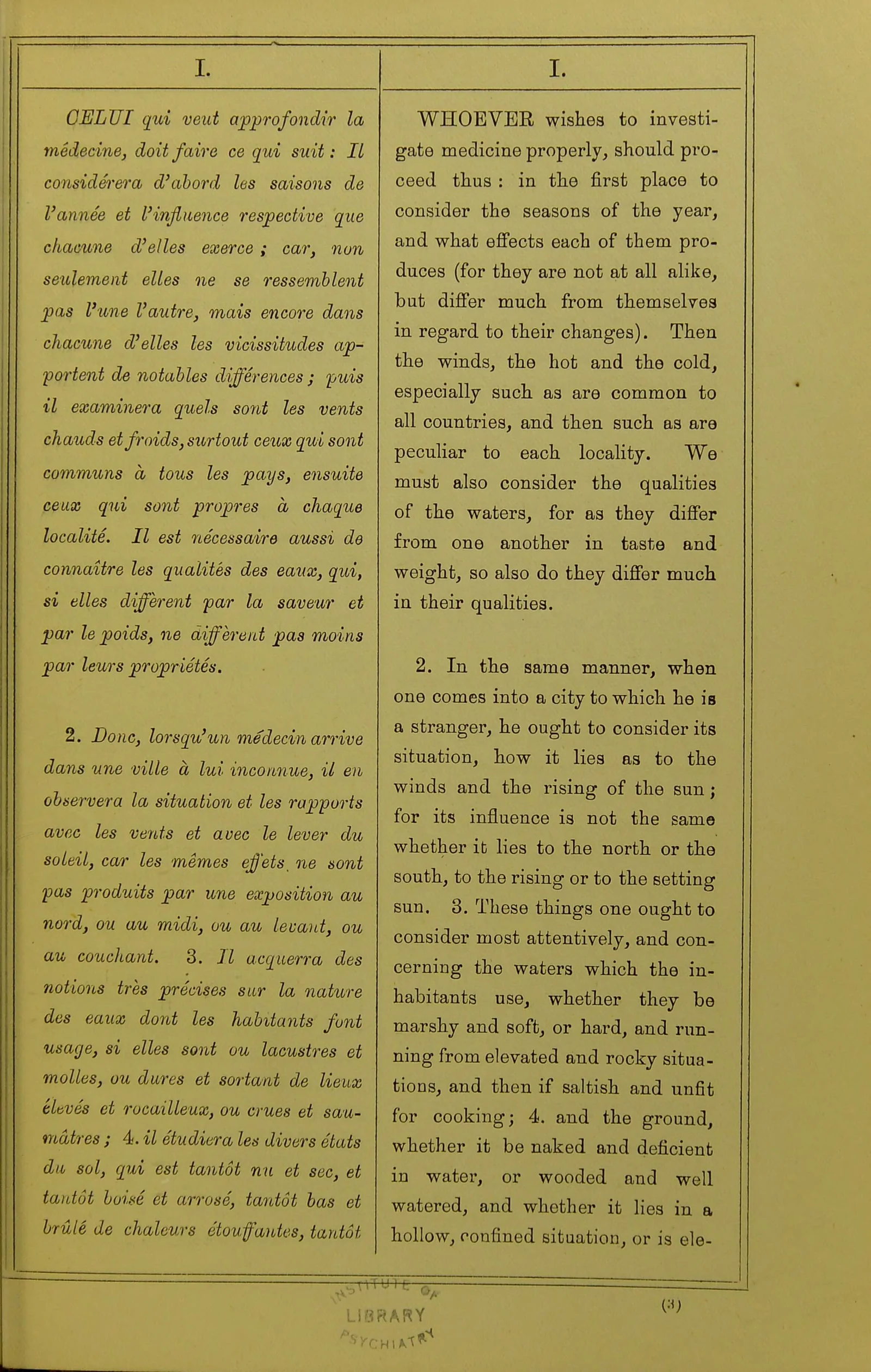
Hippocrates on airs, waters, and places: the received Greek text of Littré, with Latin, French, and English translations by eminent scholars. Image courtesy of the Wellcome Collection.

From his belief that ‘putrefaction [will] congregate with Aire’, Bacon viewed the quality of ‘aire’ as critical in the causation/transmission of disease:

“Inequalitie [imbalance, a marker of poor quality seemingly due to contamination with ‘Spirits of Bodies’ that drive ‘putrefaction’] of Aire is ever an Enemy to health”^5^ ^p. 173^

Bacon makes it clear that the purpose of his ‘noble experiments' is to discover locations where air is more ‘wholesome’ or ‘healthfull’,^5^ ^p. 204^ all with a view to identifying the most suitable ‘seats of dwelling’:

“There would be much diligence, in the Choice of some Bodies and Places, (as it were,) for the Tasting of Aire, to discover the Wholesomenesse, or Unwholesomenesse, as well of Seasons, as of the Seats of Dwellings. It is certaine, that there be some Houses, wherein Confitures [preserved fruit, like jam], and Pies, will gather Mould, more than in Others. And I am perswaded, that a Peece of Raw Flesh, of Fish, will sooner corrupt in some Aires, than in Others. They bee noble Experiments, that can make this Discovery… Better than the Astronomers can by their Figures [i.e. charts]; And againe, they teach Men to chuse their Dwelling, for their better health”^5^ ^p.164^

## ‘Experiments… [to] teach men where to chuse their Dwelling, for their better health’

He then makes it clear that the purpose of his experiments is to seek more healthy locations for what today would amount to residential homes, hotels and health resorts. Here he also alludes to his belief that the healthfulness of ‘aire’ could change from year to year. It appears that Bacon was seeking locations where the ‘aire’ was healthy year-on-year:

“As the Discoverie of the Disposition of the Aire, is good for the Prognosticks of Wholesome, and Unwholesome Years; So it is of much more use, for the Choice of Places to dwell in: At the least, for Lodges, and Retiring Places for Health; (For Mansion houses respect Provisions [i.e. stored foodstuff], as well as Health;) Wherein the Experiment [described below] may serve”^5^ ^p. 173^

Bacon's experiments call for the use of ‘flesh’ to compare speed of ‘putrefaction’ in different settings. The initial locations he proposes are inside a house and in the ‘open aire’. He considered the air in these settings to possess distinctly differing qualities in terms of putrefaction of meat. Again, he saw strong parallels in putrefaction between storage of food (‘Mansion house…provisions’) and disease causation in humans. With regard to the latter, he first calls for an experiment:

“It were good to lay a Peece of Raw Flesh, Or Fish, in the Open Aire; And if it Putrefie Quickly, it is a Sign of a Disposition in the Aire to Putrefaction. And because you cannot be informed, whether the Putrefaction be quicke or late, except you compare this Experiment with the like Experiment in another Yeare, it were not amisse in the same Yeare, and at the same Time, to lay one Peece of Flesh, or Fish, in the Open Aire, and another of the same Kinde and Bignesse, within Doores: For I judge, that if a general Disposition be in the Aire to Putrefie, the Flesh, or Fish, will sooner Putrefie abroad [i.e. outside], where the Aire hath more power, than in the House, where it hath lesse, being many wayes corrected…”.^5^ ^p. 173^

Bacon then proposes a further comparison to contrast putrefaction rates between flesh in contact with the ground and flesh suspended in the air. This hints at a four-way experiment:

“And because the Aire (no doubt) receiveth great Tincture, and Infusion from the Earth; It were good to trie that Exposing of Flesh, or Fish, upon a Stake of Wood, some height above the Earth [i.e. without touching the ground], and upon the Flat [i.e. surface] of the Earth”^5^ ^p. 173^

In the experiments above Bacon takes steps towards a fair comparison, at least in terms of ‘the same Kinde and Bignesse’ of the meat.^5^ ^p. 173^

Not least among limitations of Bacon's experiments is the challenge of identifying a precise outcome, i.e. the point of putrefaction. Nevertheless, that moment may have been much more clear-cut in an era without routine refrigeration of foods. Anyone preparing or consuming meat in the seventeenth century would possess an acute sense of smell for the moment it starts to turn bad. Other observations to herald putrefaction would be change in the consistency and colour of meat.

Bacon goes on to propose the use of ‘weather glasses’ (a basic measurement of air pressure). As is typical of Bacon, he calls for variables such as time of day, shade and cover to be controlled and outcomes (in this case condensation) to be measured:

“Inequalitie (i.e. imbalance) of Aire is ever an Enemy to health; it were good to take two Weather-Glasses, Matches in all things, and to set them, for the same Hours of One day, in severall places, where no Shade is, nor Enclosures: And to marke when you set them, how farre the Water commeth; And to compare them, when you come again, how the Water standeth then”^5^ ^p. 173^

## Bacon's wider enquiry into putrefaction

Critics will rightly find flaws in his method in these experiments, including the lack of an independent intervention. Nevertheless, it is clear that Bacon observed diverse variables, presumably at other times experimentally. In the examples below, he considers other variables in the context of ‘bodies’, here indicating foodstuffs:

“Great Heat keepeth Bodies [meat and drink] from Putrefaction; But a Tepide heat enclineth them to Putrefaction… So we find that Salt water corrupteth not so soon as Fresh… It would be tried also, whether Chalke put into Water…. doth not preserve it from Putrefying… So wee see that Strong Beere will last longer than Small [i.e. weak]”^5^ ^p. 76^

Bacon states that these factors are ‘a great Part of Physick’^5^ ^p. 75^ but defers their consideration with regard to disease under the theme of ‘ Medicinall Experiments of all Sorts’.^5^ ^p. 75^ He does, however, offer parallel explanations for successful mummification of bodies in ancient Egypt. These measures include the following:

- Removal of ‘Parts aptest to corrupt’, i.e. ‘braines’ and entrailes’,^5^ ^p. 162^
- Exculsion of ‘aire’^5^ ^p. 162^
- That the preserved body be not ‘that grosse [i.e. large]’^5^ ^p. 162^
- That it not be touching (‘commateriall’ with) another body^5^ ^p. 162^
- If the body be of ‘bulke, as a corpse is’ its surroundings must ‘drie the Mositure of the Inward Body’ as did the ‘folds of linnen, besmeared with Gums’ of Egyptian embalmers^5^ ^p. 163^

In line with his rallying call to reform learning, Bacon shows his dismay over post-mortem practice and interpretation of findings. These are revealed in his 1605 book entitled ‘Of the Proficience and Advancement of Learning, Divine and Human’ (see Figure 3):

“where as now upon opening of bodies, they [clinical findings] are passed over slightly and in silence [without sharing, presumably meaning without documentation]”^1^ ^p. 123^

**Figure 3.**
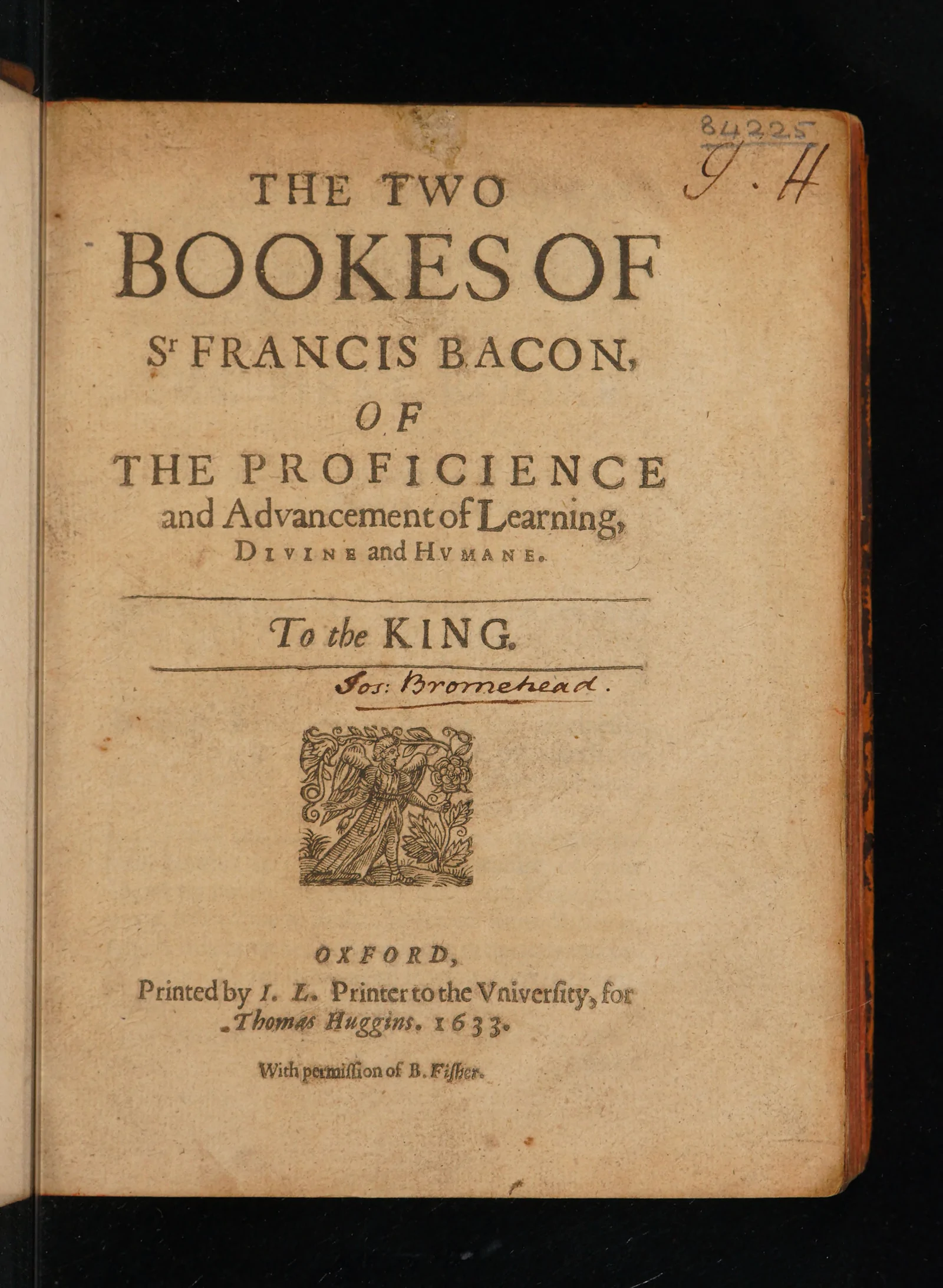
The two bookes of Sr Francis Bacon, of the proficience and advancement of learning, divine and humane. Printed in 1633 at Oxford by I. L[ichfield for Thomas Huggins]. Image courtesy of the Wellcome Trust.

He then proceeds to propose means for new learning from post-mortem examinations. First, he calls for astute observation during post-mortem examination of the nature and accumulation of different fluids: ‘what cavities, nests, and receptacles [contain] the humours’.^1^ ^p. 123^ This is important because Bacon believed that conditions like ‘Ague’ (fevers) were associated with the ‘putrefaction of humours’.^5^ ^p. 74^ He also calls for detailed consideration of wider pathological findings, including sites of ‘putrefaction’:

“And as for the footsteps [signs] of diseases, and their devastations of the inward parts, impostumations, exculcerations, discontinuations, putrefactions, consumptions, contractions, extensions, convulsions, dislocations, obstructions, repletions, together with all preternatural substances, as stones, carnosities excresences, worms, and the like”^1^ ^p. 123^

He then calls for repeated observation and combination of findings from different physicians. Those findings, Bacon insists, must be written down according to not only what is observed on post-mortem examination but also by the patient's preceding symptoms, clinical signs and diseases:

“they ought to have been exactly observed by the multitude of anatomies [i.e. many post-mortem cases], and the contributions of mens several experiences, and carefully set down, both historically, according to the appearances, and artificially, with a reference to the diseases and symptoms which resulted from them, in case where the anatomy is of a defunct [i.e. dead] patient”^1^ ^p. 123^

Bacon also highlights a need for learning from ‘anatomia comparata’. He makes it clear, however, that he also means morbid anatomy, i.e. pathology:

“There is no doubt but the fracture or framing of the inward parts is as full of difference [i.e. abnormality] as the outward, and in that is the cause continent of many diseases…”^1^ ^p. 122^

Bacon then goes on to call for comparative (i.e. animal) enquiries. He recognises both the scientific value and inhumanity of undertaking ‘dissection of beasts alive’:

“And for the passages and pores… the more subtile of them appear not in anatomies, because they are shut and latent in dead bodies, though they be open and manifest in live: which being supposed, though the inhumanity of anatomia vivorum was by Celsus justly reproved; yet in regard of the great use of this observation, the inquiry needed not by him so lightly to have been relinquished altogether, or referred to the casual practices of surgery, but might have been well diverted upon the dissection of beasts alive, which, not withstanding the dissimilitude of their parts, may sufficiently satisfy this inquiry”^1^ ^p. 122^

## Legacy of Bacon's experiments

Bacon's comparison of putrefaction rate under different environmental circumstances offers what for his day was a pioneering experimental model to calculate post-mortem interval (PMI, i.e. time passed since death).^6–9^ Bacon's observations anticipate the experiments of Schwann in 1837 into the impact of heat and alcohol on putrefaction.^10^ Bacon's observation about ‘great heat’ aligns closely with modern understanding of post-mortem decomposition. That is because excessive heat kills (or arrests the multiplication of) certain bacteria and retards their proteolytic activity.

Research into rates of post-mortem decomposition is essential to the academic field of forensic pathology in estimating likely time since death.^6–9^ Other than noting that Bacon called for ‘attention to pathological changes’,^2^ his contributions in this area appear to have been overlooked by the academic field of forensic medicine.^11^ This is probably due to their simplicity, age, and old-fashioned language.

Bacon's experiments also constitute an early search for controlled evidence to inform disease prevention through better planning of housing. Other than recognition that Bacon undertook experiments into the ‘wholesomeness’ of air for housing,^12^ the comparative, potentially four-way nature of his ‘putrefaction’ experiments appears to have been overlooked in the modern literature of public health. That is surprising because considerable effort was expended in subsequent centuries to further Bacon's enquiry into the quality of air and its impact upon ‘putrefaction’.

Most notably, the legacy of Bacon's enquiry is central in the influential works of Sir John Pringle (1707–1782), the father of military medicine. Pringle sets out his inspiration for his classic treatise (see Figure 4) thus:

“Two circumstances induced me to prosecute this subject; the great number of putrid cases that were under my care in the hospitals abroad; and the authority of Lord BACON, who offers good reasons for considering the knowledge of what brings on, and what retards, putrefaction, as most likely to account for some of the more abstruse operations of nature. My experiments on this subject have been read at several meetings of the Royal Society….”^13^

**Figure 4.**
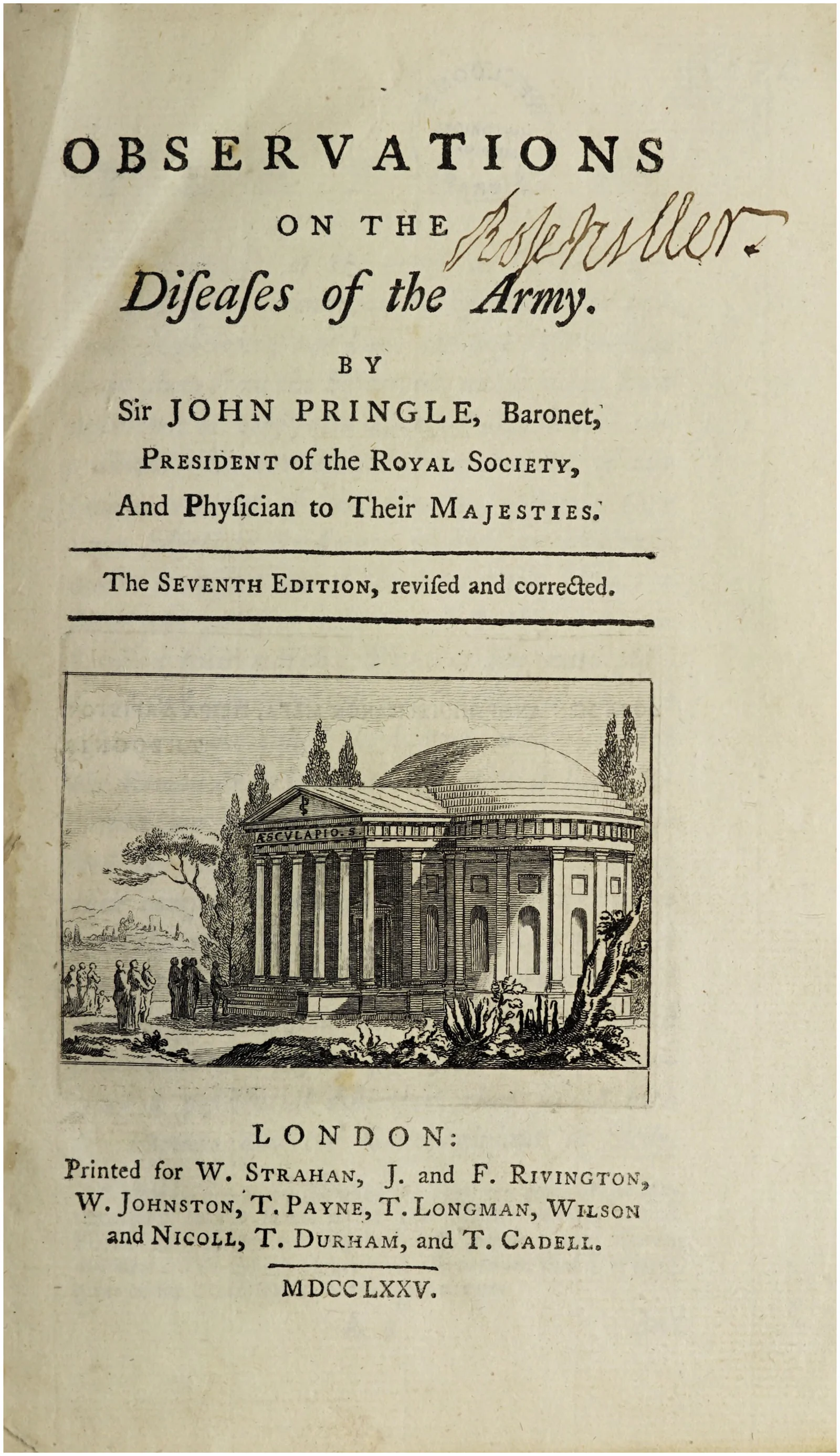
Observations on the diseases of the army, in camp and garrison (1752) by by Sir John Pringle (1707–1782). Image courtesy of the Wellcome Trust.

Contaminated air continued to be blamed for ‘putrid’ disease, by the transmission of organic matter. This is evident in the following example relating to the cause of death among anatomists in 1782 in London:

“How often do we find people infected with diseases, by only breathing the air contained in a sick person's chamber? how frequent do putrid fevers, and even consumptions arise, from inhaling air impregnated with the effluvia of putrid bodies? three fatal instances of this last kind, have happened, within these few years to three anatomical professors in London: all young men of the most promising genius”^14^

These ideas remained influential in town planning and the consideration of certain locations as being particularly favourable for health. They also formed the scientific basis of attempts at sanatory reform in the Victorian era. This is evident in the following description of the town of Brighton on the South coast of England from 1840:

“The climate of Brighton, as it affects the health of the inhabitants, may be stated as decidedly salubrious at all seasons of the year. In the autumn and winter, it is preferable to most places. It enjoys the natural advantages of constant ventilation, good surface drainage, and an absorbent and permeable soil. Neither stagnant air, nor stagnant water is to be found within or near its precincts”^15^

In the nineteenth century, these ideas were seminal in reform of living conditions. Measures taken at that time included eradication of ‘back-to-back’ and cellar dwellings^16^ and other improvements in sanitation. Bacon's notion of putrefaction transmitted by the air was, of course, to be superseded in the mid-nineteenth century by the advent of germ theory. Nevertheless, it is of historical note that the surgeon Joseph Lister described this new concept in 1865 as ‘the germ theory of putrefaction’.^17^

Bacon's ideas remain relevant today. This is most evident in the ongoing crisis in the quality of housing:

“[in 2023 in the UK] up to 2.6 million people aged 18–34 were living in poor-quality housing – defined as homes that were not in a good state of repair, where heating, electrics or plumbing were not in full working order and where damp was present”^18^

## Implications for clinical education: a need to nurture the ‘medical detective’

Bacon's enquiring mind offers an early template for research into the causes of circumstances that shape people's health. His structured enquiry ascends from ‘ordinary’ to ‘ordered experience’^19^ and has been called ‘exploratory experimentation’.^12,20^ Bacon's investigation into ‘putrefaction’ and ‘aires’ illustrates the steps inherent in his approach to ‘new learning’: astute observation, controlled comparisons, basic experiments, inductive reasoning and accurate record-keeping. Bacon's overarching philosophy towards medicine calls for a ‘forensic approach’ to learning that has implications for modern medical education.

Bacon's elegant simplicity stands in contrast to modern medical education which increasingly prioritises swift recourse to technological solutions, e.g. sequential blood tests. The end result of the latter risks emerging as a defensive, arms-length approach of ‘medicine by numbers and medicine by scans'. Conversely, interpreting Bacon's method as a ‘forensic approach’ highlights a need to (re-)kindle the natural curiosity and imagination of medical students by inspiring the ‘medical detective’ (See Table 1).

**Table 1.** Characteristics of Bacon's ‘forensic approach’ and strategies for ‘new learning’ to inspire students towards becoming the ‘medical detective’.

Philosophy
Upstream thinking Generalist ‘whole-patient’ lens Community (especially rural) perspective Valuing ‘low-level’ findings Recognition of physical and social environment in causation of illness Challenging assumptions
Process
Familiarity with ‘normal’ Astute observation Small findings may be pivotal Recognition of ‘abnormal’ and paradoxes Simple experimentation, e.g. controlled comparisons Inductive reasoning Accurate record-keeping
Inspiration
Apprenticeship model of learning 'Shoe leather’ medicine: visiting patients’ neighbourhoods (preferably on foot) and consulting inside homes Clinical experience overseas or with other cultures, i.e. where differences or contrasts may stand out Learning from ‘comparative’ contexts, e.g. veterinary medicine and global health Stories and lives from the history of medicine Wider humanities, especially literature

A further legacy of Bacon lies in the importance of clinical generalism to new learning. From the great array of his scientific interests, Bacon emerges as a true generalist. Basic observation is particularly important for generalists, especially in community-based medicine. It is in general practice (i.e. ‘family medicine’) that students become familiar with ‘normal’, i.e. patients whose presentations do not indicate serious disease.^21^ Only with a clinical foundation in the wide range of ‘normal’ is it possible to recognise ‘abnormal’ and paradoxical events. Without experience in community generalism (especially *rural* clinical practice), young doctors are likely to resort to a technological quest for diagnoses in patients one and all.

Finally, is the importance of considering the impact of patients’ ‘seats of dwelling’ upon their health. The consequences of poor-quality housing will remain invisible to students and doctors who rarely visit patients in their own homes. By visiting patients’ homes and neighbourhoods, clinicians can witness first-hand the impact of poor housing on physical and mental health. This is as valid today as it was in Victorian London:

“[Joseph Rogers (1821–1889), physician and medical officer] set up practice in Dean Street, Soho, a neighbourhood of mixed trades and fortunes [in c1844]. While caring for a patient with blood poisoning he noticed that the patient's living room wall ‘was exuding a highly putrid fluid.’ Rogers discovered that it formed part of the churchyard wall of St Anne's, Soho…”^22^

The reasons for a significant reduction in ‘shoe-leather medicine’^23,24^ (i.e. visits by doctors to patients’ homes and neighbourhoods) lie in financial efficiency, increasing reliance upon clinical tests and an expansion in digital ‘remote’ consultations. The negative impact of this decline should also be taken into account for it is within patients’ homes and neighbourhoods that doctors and students can best hone their skills of observation and induction.^24^ This is especially true in diverse cultures and countries where significant differences or contrasts may stand out.

## Conclusions

Bacon's experiments described above belong to their own day and, judged through twenty-first century eyes, have obvious conceptual flaws. Despite that, his contribution to evidence-based public health planning of housing and the understanding of post-mortem change deserve recognition in modern times. They stand as a reminder that effective public health planning and post-mortem interpretation must begin with careful observation and basic experimentation. This is in stark contrast to precedence granted by modern forensic medicine to molecular techniques, such as DNA analysis, rather than basic observational evidence.

Throughout his writing, Bacon called for the reform and advancement of learning – which he felt had stagnated. That rallying call finds resonance today in the need to inspire within medical students the art of the ‘medical detective’, i.e. familiarity with ‘normal’ signs and symptoms, attention to observation, recognition of paradoxes, natural experiments, simple experimentation, inductive reasoning and accurate record-keeping (see Table 1). This involves ‘upstream’ thinking about the role of the patient's socio-economic and physical circumstances in the causation of infectious and non-infectious disease, including mental health conditions.

Bacon's ‘spirit’ of forensic enquiry risks being eclipsed in undergraduate medical education by today's ‘law’ of technological, diagnostic and translational medicine. This loss forms part of a transition from Bacon's ‘new learning’ to corporate ‘credentialing’^25^ in higher education over the past few decades. Central to this process is the assessment of medical students through factual ‘single best answer’ (i.e. multiple choice) questions^26^ rather than supportive apprenticeship learning in clinical practice.

Promoting the ‘medical detective’ in education calls for clinical teachers to (re-)kindle students’ own imagination and naturally inquisitive minds in the following ways. First, is by consulting inside patients’ ‘seats of dwelling’ and, ideally, travelling on foot in their neighbourhood. This may be particularly powerful in diverse countries or cultures. Second, is through the study of the history of medicine, not least the incremental advances of inspirational and generalist researchers such as Lord Francis Bacon.

Finally, is by studying the wider humanities, i.e. beyond textbooks of factual medicine. Here students’ imagination can be awakened to new ideas and the spirit of enquiry. As an example, one need look no further than Shakespeare. In his play Hamlet – at the moment of discovering the skull of Yorick, the King's jester – Shakespeare's pen turns to the very subject of post-mortem interval:

“HAMLET: How long will a man lie i’ th’ earth ere [before] he rot? GRAVEDIGGER: Faith, if he be not rotten before he die (as we have many pocky cor[p]ses nowadays that will scarce hold the laying in), he will last you some eight year or nine year. A tanner will last you nine year.

HAMLET: Why he more than another?

GRAVEDIGGER: Why, sir, his hide is so tanned with his trade that he will keep out water a great while; and your water is a sore decayer of your whoreson dead body”^27,28^
